# Uremic Toxins and Atrial Fibrillation: Mechanisms and Therapeutic Implications

**DOI:** 10.3390/toxins11100597

**Published:** 2019-10-13

**Authors:** Fumi Yamagami, Kazuko Tajiri, Dai Yumino, Masaki Ieda

**Affiliations:** 1 Department of Cardiology, Faculty of Medicine, University of Tsukuba, Tsukuba, Ibaraki 305-8575, Japan; fumya822@gmail.com (F.Y.); mieda@md.tsukuba.ac.jp (M.I.); 2 YUMINO Heart Clinic, Toshima-ku, Tokyo 171-0033, Japan; yumino@yumino-clinic.com

**Keywords:** atrial fibrillation, uremic toxin, indoxyl sulfate, chronic kidney disease

## Abstract

Atrial fibrillation (AF) is the most prevalent arrhythmia in the general population. There is a close association between chronic kidney disease (CKD) and AF. In recent years, attention has been focused on the relationship between AF and uremic toxins, including indoxyl sulfate (IS). Several animal studies have shown that IS promotes the development and progression of AF. IS has been shown to cause fibrosis and inflammation in the myocardium and exacerbate AF by causing oxidative stress and reducing antioxidative defense. Administration of AST-120, an absorbent of uremic toxins, decreases uremic toxin-induced AF in rodents. We have recently reported that patients with a higher serum IS level exhibit a higher rate of AF recurrence after catheter ablation, with serum IS being a significant predictor of AF recurrence. In this review, we discuss the possible mechanisms behind the AF-promoting effects of uremic toxins and summarize the reported clinical studies of uremic toxin-induced AF.

## 1. Introduction

Atrial fibrillation (AF) is a common and chronic cardiovascular condition. AF requires expensive health monitoring and treatment because AF patients have an increased risk of stroke, sudden death, heart failure, unplanned hospital admissions, and other complications [1,2,3,4]. Although the management of AF has substantially advanced, with significant developments in the past few decades, the contributing factors and mechanisms promoting AF are still unclear.

An association between chronic kidney disease (CKD) and AF has been described, and several studies have revealed the complex relationship between CKD and AF. In this review, we focused on the causative association between uremic toxins and AF.

## 2. Epidemiology of AF

AF is the most prevalent arrhythmia in the general population [1] and is associated with an increased risk of stroke [2], heart failure [3], and mortality [4]. The number of people with AF has been increasing worldwide because of an aging general population, and an increased incidence of AF is expected with the increasing life expectancy of society [1,5]. The Global Burden of Disease Study in 2010 [6] stated that the age-adjusted mortality rate (per 100,000 population) for AF patients in 1990 was 0.8 for men and 0.9 for women. The age-adjusted mortality rate had increased to 1.6 and 1.7 by 2010, representing a 2-fold and 1.9-fold increase for men and women, respectively. In 1990, the overall incidence rates of AF in the world population were 60.7 per 100,000 person-years in men and 43.8 in women. In 2010, the estimated incidence rates had increased to 77.5 per 100,000 persons-years in men and 59.5 in women. It is predicted that AF will affect 5–16 million people in the United States and more than 1 million people in Japan by 2050 [5]. Therefore, AF is one of the most important cardiovascular diseases that needs to be effectively managed in aging populations.

## 3. Pathophysiological Mechanisms of AF

### 3.1. Triggers and Substrate of AF

AF is a supraventricular tachyarrhythmia characterized by uncoordinated atrial activation with the subsequent deterioration of atrial mechanical function. It is well accepted that AF development needs both a trigger and a susceptible substrate. AF is produced by triggered activity from pulmonary vein ectopic foci or the genesis of re-entry circuits in atrial substrates [7], and sustained high frequency reentrant AF drivers (rotors) can be produced by a focal trigger. The electrical waves emerge from the rotors and undergo spatially distributed fragmentation and give rise to fibrillatory conduction [8].

### 3.2. Cardiac Autonomic Nervous System and Triggered Spontaneous Pulmonary Vein Firing

Autonomic nerve input to the atria originate from both the central autonomic (preganglionic) nervous system and the intrinsic cardiac autonomic nervous system. The intrinsic cardiac autonomic nervous system contains autonomic ganglionated plexi, or clusters of ganglia. The autonomic ganglionated plexi receive input from the central extrinsic autonomic nervous system and numerous interconnecting neurons that provide communication within and between the ganglionated plexi. Focal firing in the pulmonary veins by ganglionated plexi stimulation requires both sympathetic and parasympathetic activity. Parasympathetic stimulation shortens the action potential duration and effective refractory period in atrial and pulmonary vein myocytes, and sympathetic stimulation increases calcium loading and automaticity. Ganglionated plexi may promote AF by activating triggered spontaneous pulmonary vein firing [8].

## 4. Treatment of AF

The three domains of AF management are (1) the stabilization of underlying and accompanying cardiovascular conditions, (2) stroke risk assessment and oral anticoagulation for stroke prevention, and (3) heart rate and rhythm control therapy [9].

### 4.1. Stabilization of Underlying and Accompanying Cardiovascular Conditions

The presence of AF strongly indicates that other cardiovascular comorbidities are present, such as hypertension, heart failure, valvular heart disease, obesity, and coronary artery disease. Focusing on heart failure, it is well known that AF increases the risk of stroke, hospitalization, and death in heart failure patients. The treatment of AF can substantially alter long-term outcomes in patients with heart failure. In addition, heart failure may promote AF by creating an AF-promoting atrial substrate via left atrial dilatation, activating the cardiac autonomic nervous system and inducing a focus of inflammation [10].

### 4.2. Stroke Risk Assessment and Oral Anticoagulation for Stroke Prevention

AF patients have a hypercoagulable state, and AF is one of the most important causes of thromboembolism. The presence of AF is an independent risk factor for stroke and thromboembolism, and stroke associated with AF increases mortality and morbidity [11]. Patients with one or more stroke risk factors (CHA2DS2-VASc score of ≥1) should be treated with oral anticoagulants such as well-controlled warfarin or a direct oral anticoagulant.

### 4.3. Rate and Rhythm Control Therapy

In AF, each part of the atrial myocardium is stimulated at a rate of 300–400 activations per minute. The rapid reactivation of atrial myocardium leads to a cessation of atrial contractility and results in rapid and irregular ventricular rates. Heart rate control is achieved pharmacologically by slowing the atrioventricular nodal conduction using the β-blockers digoxin or digitoxin, as well as verapamil or diltiazem. Rhythm control with antiarrhythmic drugs are not superior to heart rate control in patients with coexisting heart failure and AF [12]. Another way to maintain sinus rhythm is through catheter ablation, which is a well-established option for drug resistant symptomatic AF in patients with otherwise normal cardiac function [13,14,15,16,17] and in patients with heart failure [18,19,20,21,22]. Moreover, a recent study has shown that catheter ablation is superior to drug therapy for AF patients with heart failure, resulting in an improvement in left ventricular ejection fraction, quality of life, functional status, and mortality [23,24,25,26]. Although catheter ablation has become an established treatment option as implemented in the current guidelines for the treatment of AF [17,27], post-procedure AF recurrence remains a major clinical problem. Unfortunately, the mechanisms of AF recurrence after ablation are unknown. Patient selection seems to play an important role in the procedural success and risk stratification for the prevention of AF recurrence. This is based on pre-existing clinical patient characteristics, such as age, the pattern and duration of AF, and the degree of atrial enlargement. However, these indicators are insufficient to predict AF recurrence [28]. Therefore, new AF biomarkers are needed to predict treatment response after catheter ablation and better understand the mechanisms that promote AF development and recurrence.

## 5. AF and CKD

Several clinical trials have shown that AF and CKD are closely related [2,29,30,31,32]. It has been reported that the prevalence of AF in CKD patients is 2–3 times higher than that in the general population [33,34]. Moreover, the prevalence of AF in dialysis patients is as high as 27% [35,36,37,38], compared to 1.0% in the general population [16], indicating that dialysis cannot reduce the risk of AF. Therefore, non-dialysis uremic factors are thought to be factors that promote atrial fibrillation. Uremic cardiomyopathy is a distinctive type of heart failure associated with CKD. Uremic cardiomyopathy is caused by an impairment of microvascular function, low-grade inflammation, oxidative stress, and enhanced cardiac fibrosis. In addition, hypertension, anemia, and activation of the renin–angiotensin–aldosterone system and sympathetic nervous may contribute to the occurrence of uremic cardiomyopathy in CKD. The pathophysiological effects of CKD contribute multiple arrhythmogenic factors to the development of AF [39,40,41].

In addition, the relationship between AF recurrence after catheter ablation for AF and CKD has been addressed in recent reports [42,43,44]. Patients with CKD were at high risk of AF recurrence after catheter ablation. Although atrial remodeling associated with renal dysfunction was thought to be responsible for the poor prognosis after AF ablation in CKD patients, the exact mechanism has not been fully elucidated.

## 6. Uremic Toxins and Cardiovascular Diseases

Protein-bound uremic toxins, such as indoxyl sulfate (IS), indole-3 acetic acid (IAA), *p*-cresol, and *p*-cresyl sulfate, which originate from protein fermentation, can increase oxidative stress, inflammation, and activate the neurohormonal system that results in cardiovascular fibrosis and oxidative injury [45,46]. Furthermore, uremic toxins produce pro-hypertrophic, pro-inflammatory, pro-fibrotic conditions in cardiomyocytes [47,48,49].

IS is a uremic toxin that has high protein-binding ability and is poorly dialyzable. Even after hemodialysis, the serum IS level remains high [50]. IS is one of the most common uremic toxins derived from dietary protein metabolism by the gut microbiota and is involved in the pathogenesis of cardiovascular diseases including AF [51,52,53,54]. Recently, the relationship between IS and cardiovascular diseases among CKD patients is attracting increasing attention. Barreto et al. reported that an elevated serum IS level was associated with an increased overall death and cardiovascular-related death among CKD patients [55]. Furthermore, Lin et al. [56] indicated that a serum IS level was a useful biomarker in predicting cardiovascular events in advanced CKD patients [56]. Other researchers have reported an association between elevated IS levels and an increased risk of left ventricular diastolic dysfunction [57,58].

Although IAA has not been studied as much as IS, a recent study suggests that IAA has a cardiovascular toxic effect and is associated with the progress of cardiovascular disease [53]. Dou et al. [53] studied patients with CKD and found that mortality and cardiovascular events were significantly higher in the higher IAA group (IAA > 3.73 mM) than in the lower IAA group (IAA < 3.73 mM). The IAA concentration positively correlated with malondialdehyde and C-reactive protein (CRP) was used to evaluate oxidative stress and inflammation. In multivariate Cox regression analysis, serum IAA was a significant predictor of mortality and cardiovascular events even after adjustments for CKD stage [53]. They also demonstrated direct effects of IAA on endothelial cells. In culture experiments with human endothelial cells, IAA activated an inflammatory nongenomic aryl hydrocarbon receptor (AhR)/p38 MAPK/NF-kB pathway involved in the pro-inflammatory enzyme cyclooxygenase-2 upregulation. Moreover, IAA increased production of reactive oxygen species from the endothelial cells. Thus, IAA has prooxidant and pro-inflammatory effects in human endothelial cells, which may explain the association between IAA concentration and the increased risk of mortality and cardiovascular events in CKD patients [53].

Acute kidney injury (AKI) is also a known risk factor for AF [59] and IS is associated with higher mortality in AKI patients [60]. Thus, therapies targeting both AKI and IS are needed [61].

## 7. IS and AF

In recent years, attention has been focused on the relationship between AF and uremic toxins, especially IS. Several animal studies have revealed that IS has a causative role in exacerbating AF. IS has been shown to promote AF via its effect on cardiac fibrosis and inflammation by increasing oxidative stress; the administration of AST-120, an absorbent of uremic toxins, decreased AF inducibility in rodents [62,63]. In the clinic, we have recently reported that patients with elevated serum IS levels showed a higher AF recurrence rate after successful catheter ablation, with serum IS being a significant predictor of AF recurrence [64].

### 7.1. Experimental Studies in Animal Models

**Figure 1:**
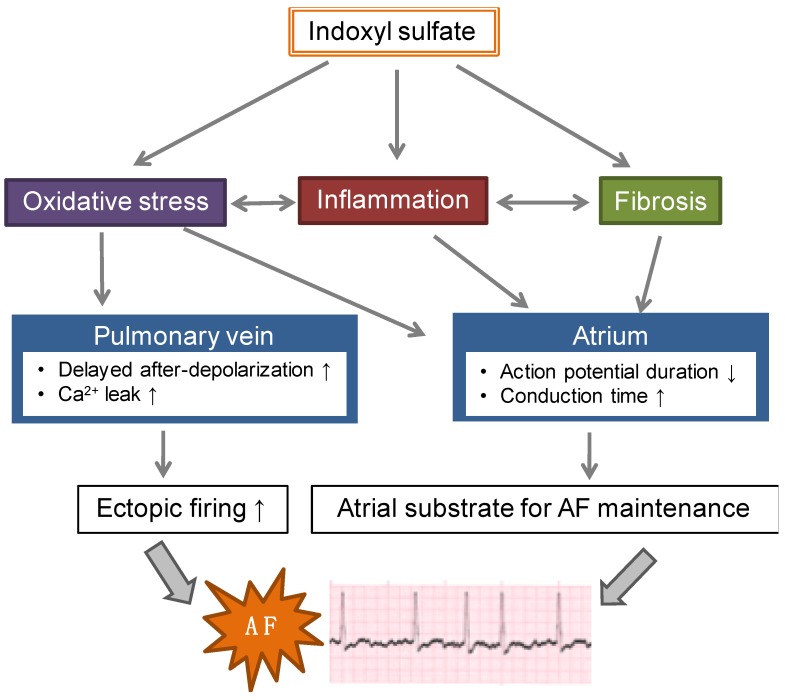
Role of indoxyl sulfate (IS) in the progression of atrial fibrillation (AF). IS induces oxidative stress and promotes arrhythmogenesis in the pulmonary vein and atrium. In the pulmonary vein, IS induces an increased occurrence of delayed after-depolarizations, burst firing, and increased calcium leakage. In the atrium, IS shortens the action potential duration. IS also induces inflammation and fibrosis in the atrium, which leads to conduction time prolongation. These IS effects on the pulmonary vein and atrium exacerbates the development of AF substrates through increased ectopic firing and re-entry circuits in atrial substrates.

The arrhythmogenic effects of IS have been demonstrated in in vitro, ex vivo, and in vivo experiments (Figure 1). Chen et al. [63] showed that IS induced an increased occurrence of delayed after-depolarizations, burst firing, and increased calcium leakage in isolated pulmonary veins, and decreased spontaneous beating of the sinoatrial nodes and shortened the action potential durations in left atria isolated from rabbits. Stimulation with burst pacing and isoproterenol (a β-agonist) induced an increased occurrence of AF and a longer AF duration in the left atrial tissue with IS than without IS. This IS-induced arrhythmogenesis was attenuated by the antioxidant ascorbic acid. These data suggested that IS increases pulmonary vein and atrial arrhythmogenesis through an oxidative stress-dependent mechanism.

Lekawanvijit et al. demonstrated direct effects of IS on cardiac fibroblasts and myocytes [47]. Stimulation with IS in neonatal rat fibroblasts and cardiomyocytes increased collagen synthesis and myocyte hypertrophy, respectively. They also showed that proinflammatory effects of IS in cultured THP-1 cells, a human monocytic cell line, determined by a significant increase in inflammatory cytokines tumor necrosis factor-alpha, interleukin-6 (IL-6), and IL-1β. Thus, IS may have pro-fibrotic, pro-hypertrophic, and pro-inflammatory effects on cardiac cells.

In a rat model of CKD induced by 5/6 nephrectomy, Aoki et al. demonstrated that IS increases the occurrence of AF in vivo [62]. Serum IS level was significantly increased after 5/6 nephrectomy. In electrophysiological experiments, AF was induced by atrial extrastimuli at almost 100% of the induction rate. They showed that administration of AST-120—that is commonly used in clinical settings in Japan as an absorbent of uremic toxins to delay incident renal replacement therapy—decreased the serum level of IS, resulting in decreased oxidative stress, reduced inflammation, reduced fibrosis in the left atrium, and decreased occurrence of AF. Incubation of cultured atrial fibroblasts with IS upregulated the expression of NADPH oxidase 2 and 4 and malondialdehyde (oxidative stress markers), along with an increase in profibrotic and inflammatory molecules, such as α-smooth muscle actin, transforming growth factor β1, collagen type 1, and monocyte chemoattractant protein 1 (MCP-1), [62]. These data suggested that IS could have a pathogenetic factor for AF in renal dysfunction mediated by the progression of atrial remodeling by oxidative stress, fibrosis, and inflammation.

### 7.2. IS and AF Recurrence after Catheter Ablation

**Figure 2:**
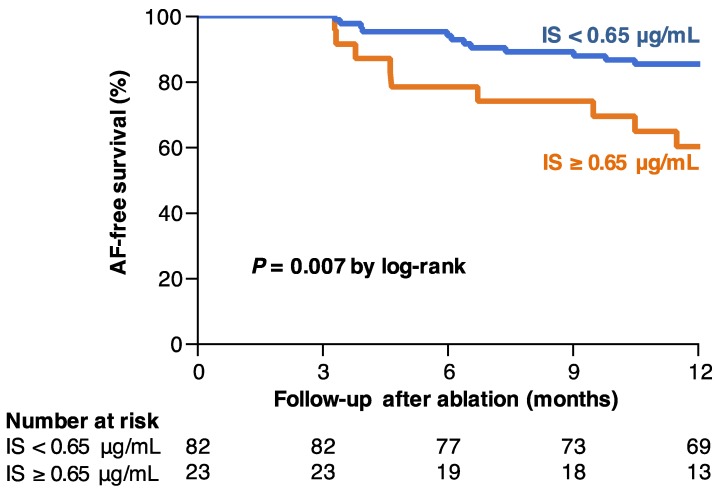
Impact of indoxyl sulfate (IS) levels on the atrial fibrillation (AF) recurrence in patients undergoing radiofrequency catheter ablation. The 1-year AF-free survival is shown according to IS levels. The numbers at the bottom of the graph shows the number of ‘at risk’ patients in each follow-up month. Reproduced from [64], 2018, Springer Nature.

The high AF recurrence rate after ablation in CKD patients suggests that uremic toxins may be involved in the development of AF. Although several studies have investigated the association between IS and cardiovascular disease as we described above, limited clinical data support the association between IS and AF. Therefore, we investigated the association between IS levels and AF recurrence after radiofrequency catheter ablation [64]. The study investigated 125 consecutive patients with nonvalvular AF scheduled for catheter ablation for AF. This cohort included patients with normal or mild-to-moderate reduced renal function (CKD 1–3). Serum levels of IS and IAA were measured by reversed-phase high-performance liquid chromatography before catheter ablation [65]. After catheter ablation, follow-ups were performed at 1, 3, 6, and 12 months. In this cohort, serum IS levels were significantly increased in patients with CKD stage 3 compared to CKD stage 1 and 2. On the other hand, IAA levels were not statistically different among patients with CKD stage 1, 2, and 3. The correlations of serum IS levels with estimated glomerular filtration rate (eGFR) (*r* = −0.295, *p* = 0.002) and creatinine clearance (CrCl) (*r* = −0.263, *p* = 0.007) were weak. IAA levels were also weakly correlated with eGFR (*r* = −0.255, *p* = 0.009), but not with CrCl (*r* = −0.102, *p* = 0.30). Patients were divided into two groups based on serum IS levels, the high (≥ 0.65 μg/mL) and low (< 0.65 μg/mL) IS group, which was set as the optimal cutoff value determined by the maximum Youden index (sensitivity + specificity − 1), on the basis of receiver operating characteristic curve analysis. The 1-year AF-free survival was markedly lower in patients with high serum IS levels than in those with low IS levels (60.1 ± 10.4% versus 85.2 ± 3.9%, *p* = 0.007) (Figure 2). In the univariate analysis, a serum IS level ≥ 0.65 μg/mL was associated with the recurrence of AF (hazard ratio = 3.10 [1.26–7.32], *p* = 0.015), and this association was maintained in multivariate Cox proportional hazard model (hazard ratio = 3.67 [1.13–11.7], *p* = 0.031) [64]. Thus, in patients undergoing successful catheter ablation, we identified that baseline IS levels independently predict AF recurrence.

## 8. Therapeutic Potential of AST-120 for AF

AST-120 (KREMEZIN) is approved in Japan, Korea, and the Philippines for progressive CKD patients as an absorbent of uremic toxins to delay incident renal replacement therapy in clinical settings [66]. AST-120 inhibits the absorption of indoles from the intestines and thus effectively reduces circulating and renal IS levels independent of renal dysfunction [52,67,68] AST-120 decreases serum IS levels in a dose-dependent fashion [69]. In uremic animal models, AST-120 has been shown to improve renal function and structure [68,70]. In humans, several prospective clinical trials have demonstrated the protective effects of AST-120 against the progression of renal dysfunction [71,72,73,74]. However, recent large-scale randomized controlled trials in humans have not observed any beneficial effects of AST-120 on impeding CKD progression [75,76]. There are several ongoing or unpublished clinical studies registered in ClinicalTrials.gov, which will reveal the role of AST-120 in CKD.

On the other hand, many animal studies and small human studies have suggested that AST-120 may have protective effects in cardiovascular disease. Endothelial dysfunction induced by the acute and chronic inflammatory status in CKD patients contributes towards overt cardiovascular disease [77]. There are several reports regarding the beneficial role of AST-120 on vascular function in CKD animals and patients. Treatment with AST-120 has been shown to ameliorate the following: endothelial dysfunction in CKD rats [78]; the extent and instability of atherosclerosis induced by kidney disease in apolipoprotein E-deficient mice [79]; flow-mediated vasodilation in pre-dialysis CKD patients [80]; and microvascular endothelial dysfunction and carotid arterial intima-media thickness in patients receiving hemodialysis [81]. There are also several reports supporting the beneficial effects of AST-120 in the prevention of left ventricular hypertrophy in CKD rats [82,83] and pre-dialysis patients [84]. More recently, Asanuma et al. [85] demonstrated that AST-120 treatment inhibits cardiac remodeling, attenuates apoptosis, and prevents the progression of heart failure in a dog model of heart failure induced by rapid right ventricular pacing.

There has been only one study that has examined the protective effects of AST-120 on AF [62]. Aoki et al. [62] examined the effect of AST-120 treatment in a rat model of CKD induced by 5/6 nephrectomy. AF induced by atrial extra stimuli in perfused hearts extracted from CKD rats was attenuated by AST-120 treatment. Left atrial enlargement and ventricular concentric hypertrophy were significantly prevented by AST-120 treatment, without significant improvement in systolic blood pressure and renal function [62]. AST-120 treatment suppressed expression of MCP-1 and vascular cell adhesion molecule 1 and infiltration of CD68-positive inflammatory cells in the atrium, suggesting that AST-120 attenuated monocyte-mediated inflammation in the atrium [62]. Overall, AST-120 appears to have protective effects against cardiovascular diseases including AF; however, additional randomized controlled trials are required to determine whether AST-120 reduces the risk of cardiovascular diseases in CKD patients.

## 9. Conclusions

In this review, we investigated the effects of protein-bound uremic toxins, especially IS, on the pathogenesis of AF. Using animal models, several studies have demonstrated that IS can exacerbate AF directly and indirectly by promoting enhanced oxidative stress and reduced antioxidative defense via its effect on cardiac fibrosis and inflammation. Clinical studies have supported the association between IS and AF by identifying elevated IS levels as a strong and independent predictor of AF recurrence in patients undergoing successful catheter ablation.

Methods to remove uremic toxins in the body are limited due to their high protein-binding capacity. AST-120 might be a new therapeutic compound to prevent AF and reduce AF recurrence in patients after catheter ablation.
